# Dyslipidemia in people living with HIV-AIDS in a tertiary hospital in South-East Nigeria

**DOI:** 10.11604/pamj.2017.28.204.13505

**Published:** 2017-11-06

**Authors:** Ernest Ndukaife Anyabolu

**Affiliations:** 1 Division of Nephrology, Department of Medicine, Imo State University Teaching Hospital, Orlu, Nigeria; 2 Department of Medicine, Chukwuemeka Odumegwu Ojukwu University Teaching Hospital, Awka, Nigeria

**Keywords:** Anemia, body mass index, CD4 cells count, dyslipidemia, HIV, urine protein, urine osmolality, Owerri, Nigeria

## Abstract

**Introduction:**

Across the globe, human immunodeficiency virus (HIV) infection is a healthcare problem. Dyslipidemia, a cardiovascular risk factor, is known to occur with the progression of HIV infection. The factors which influence dyslipidemia in HIV subjects have not been completely identified. The aim of this study was to evaluate serum lipids and identify the factors which might influence dyslipidemia in treatment-naïve HIV subjects in Owerri, Nigeria.

**Methods:**

This was a cross-sectional study of treatment-naïve HIV subjects. Anthropometric and demographic data were collected. Serum LDL serum cholesterol, serum high density lipoprotein cholesterol, serum triglyceride, spot urine creatinine, spot urine osmolality, spot urine protein, serum creatinine, 24-hour urine protein, 24-hour urine osmolality, 24-hour urine creatinine, creatinine clearance and hemoglobin were conducted. The variables were compared between those who have dyslipidemia and those who have no dyslipidemia.

**Results:**

The mean age of the subjects was 39 ± 11 years. Females constituted 72.0% and males 28.0%. Elevated serum LDL was present in 17.6%, elevated serum total cholesterol in 11.4%, elevated serum triglyceride in 9.9% and low serum HDL in 34.4% of the subjects. There was significant association between dyslipidemia and CD4 cells count, as well as anemia. There was no significant association between dyslipidemia and urine protein, urine creatinine, urine osmolality, creatinine clearance, as well as 24-hour urine volume.

**Conclusion:**

The prevalence of dyslipidemia was high in the study subjects. Abnormal CD4 cells count and anemia were common in treatment-naïve HIV subjects who have dyslipidemia.

## Introduction

Very overbearing has been the enormity of cardiovascular disease, with its attendant morbidity and mortality, taking a great toll on the economies of many nations [1]. Dyslipidemia is a major cardiovascular risk and a great healthcare problem in both the developed and the developing countries [2, 3]. The world prevalence of dyslipidemia in human immunodeficiency virus (HIV) subjects is not completely known. Similarly, its prevalence in Sub-Saharan African countries is also not well established, but two studies put the rate at 76.9% and 78.9% [4, 5]. Elevated serum LDL, elevated serum total cholesterol, elevated serum triglyceride and low serum HDL, in isolation or in combination and also known as abnormal serum lipids, define dyslipidemia [6-8]. Dyslipidemia, whether in combination or in isolation, has an ominous contribution to the pathogenesis of atherosclerosis [9]. Atherosclerosis commonly results in abnormal vascular thickening, loss of vascular compliance and stenosis of the affected vessels, which will progressively compromise vascular perfusion to vital organs, leading to ischemia and subsequently, compromised function of the organ. Isolated elevated serum LDL and a combination of elevated serum LDL and other abnormal serum lipids have been shown to be associated with cardiovascular disease [9, 10]. Gender, diet, age, hypertension, body mass index (BMI), diabetes mellitus, combined antiretroviral therapy (cART), among others, have been identified by some studies as the factors associated with dyslipidemia [11, 12]. There is a paucity of studies on dyslipidemia and its associated factors in treatment-naïve HIV subjects in Nigeria. As a result of this, we have embarked on this study to determine the prevalence of dyslipidemia and identify the factors, including indices of renal impairment, which may influence dyslipidemia, targeting to institute early intervention measures to whittle down adverse cardiovascular consequences in HIV subjects at the early stage of the infection.

## Methods

This was a cross-sectional study involving 393 subjects consecutively drawn from the HIV clinic in Federal Medical Centre (FMC), Owerri, South-East Nigeria. This hospital, is a tertiary health institution, second only to Imo State University Teaching Hospital, in the state. It receives referrals from the state as well as from the neighboring states. Owerri Municipal, where it situates, has a probable population of 125,337, whereas the state has about 3,927,563 people [13]. The study was conducted between April and August 2011. The inclusion criteria were treatment-naïve HIV seropositive status and age between 16 and 65 years. Those who were pregnant, or have known pituitary, adrenal, renal or terminal illness were excluded from the study. Informed written consent was obtained from each of the participants. Approval for the study was given by the Ethics Committee of the hospital, with Ethics Approval Reference Number FMC/HCS/VOL II, dated 16^th^ March, 2011. With the aid of a questionnaire, anthropometric and demographic data were obtained. Our laboratory technicians administered the questionnaire and obtained the relevant data. Because the study was hospital-based, it was not pre-tested, as data collection was not difficult. In both English and our native language, the aim of the study was explained to the subjects. The place of domicile and origin, age and gender of the subjects were obtained. Weight and height were measured and BMI taken as the ratio of weight/height^2^ (kg/m^2^). Blood pressure measurements were taken [10]. Clear instructions were given to all the subjects on how to collect 24-hour urine sample. For each subject, a day-time random spot urine sample and blood samples were collected at the end of the 24-hour urine sample collection [10]. From the random spot urine samples collected, spot urine protein (SUP), spot urine creatinine (SUCr) and spot urine osmolality (SUOsm) were performed. Also from the 24-hour urine samples collected, 24-hour urine protein (24HUP), 24-hour urine creatinine (24HUCr) and 24-hour urine osmolality (24HUOsm) were performed. Hemoglobin (Hb) and serum creatinine were performed on the blood samples collected. Other tests done from the blood samples were HIV screening and confirmatory tests, fasting and fasting serum lipid profile (FSLP) (total cholesterol, triglyceride, HDL, LDL). Osmolality was determined by freezing point depression method using Precision Osmette 5002 osmometer, creatinine by modified Jeff's method and protein by photometric method. Creatinine clearance (ClCr) was determined [14-16].

**Statistical analyses**: SPSS version 17.0 (SPSS Int. Chicago, II, USA) was used in analyzing the data. The distribution and characterization of the clinical and laboratory features among the subjects with different levels of serum LDL were analyzed using cross-tabulation. For continuous variables, mean values and standard deviations were calculated and the means compared using student t-test or two sample t-test. Categorical variables were compared using the nonparametric tests-Chi-squares. All tests were two-tailed. P < 0.05 was taken as statistically significant. The potential risk factors of dyslipidemia evaluated were SUCr, SUOsm, BMI, SCr, SUP, 24HUCr, 24HUOsm, ClCr and Hb.

**Definition of terms**: WHO classification was used to define BMI levels as follows [17]: underweight = BMI < 18.5kg/m^2^; Normal weight = BMI 18.5 - 24.9kg/m^2^; Overweight = BMI 25.0-29.9kg/m^2^; Obesity class I = BMI 30.0-34.9kg/m^2^; Obesity class II = BMI 35.0-39.9kg/m^2^; Obesity class III = BMI ≥ 40.0kg/m^2^. However, in this study, obesity was defined as class I, class II and class III obesity added together. Normal urine osmolality: 24HUOsm 300 - 750mOsm/kgH2O [18, 19]. Dilute urine: 24HUOsm < 300mOsm/kgH2O. Concentrated urine: 24HUOsm >750mOsm/kgH2O. Anemia was defined according to the WHO criteria [20]. No anemia: Hb > 13.0g/dl in males and Hb > 12.0g/dl in females. Mild anemia: Hb 11.0-13.0g/dl in males and Hb 11.0-12.0g/dl in females. Moderate anemia: Hb 8.0-10.9g/dl in males and Hb 8.0-10.9g/dl in females. Severe anemia: Hb < 8.0g/dl in males and Hb < 8.0g/dl in females. However, in this study, anemia was defined as Hb < 13.0.0g/dl in males and Hb < 12.0g/dl in females. Overall, in this study, anemia was defined as Hb ≤ 12.0g/dl. Desirable serum LDL: serum LDL < 2.6mmol/l. Borderline serum LDL: serum LDL 2.6-4.1mmol/l. High serum LDL: serum LDL > 4.1mmol/l. Desirable serum cholesterol: serum cholesterol < 5.2mmol/l. Borderline serum cholesterol: serum cholesterol 5.2-6.2mmol/l. High serum cholesterol: serum cholesterol > 6.2mmol/l. Desirable serum triglyceride: serum triglyceride < 1.7mmol/l. Borderline serum triglyceride: serum triglyceride 1.7-2.2mmol/l. High serum triglyceride: serum triglyceride > 2.2mmol/l. Low serum HDL: serum HDL < 1.0mmol/l. Desirable serum HDL: serum HDL ≥ 1.0mmol [10, 21].

## Results

**Figure 1:**
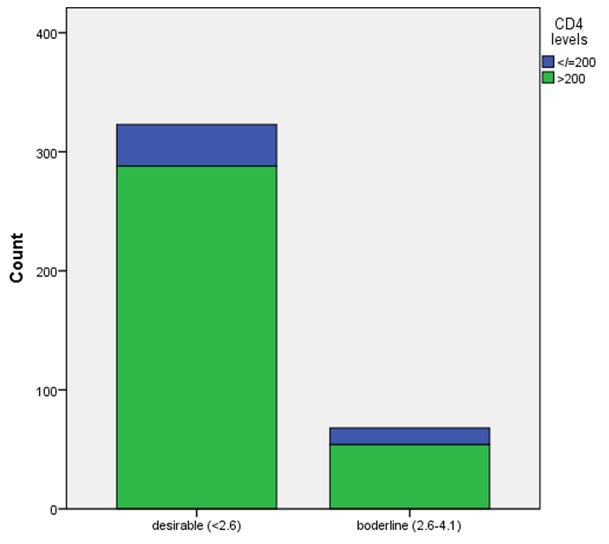
Association between LDL and CD4 cells count in treatment-naïve HIV subjects

**Figure 2:**
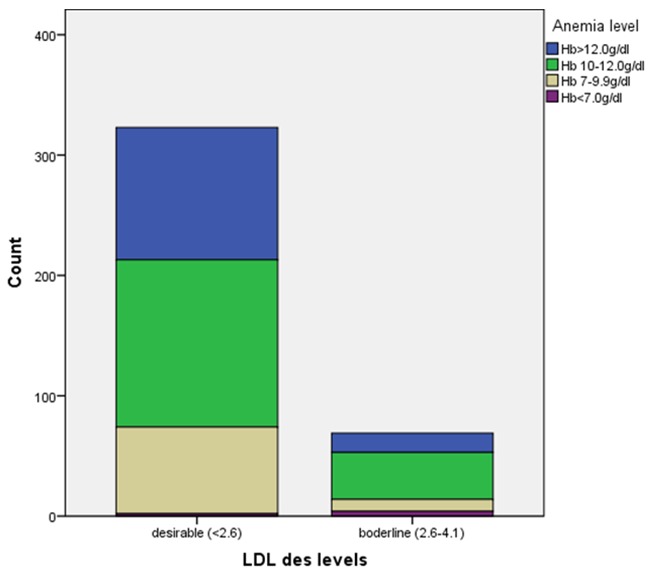
Association between LDL and anemia

This study evaluated anthropometric and biochemical profiles in 393 treatment-naïve subjects. Of these, 72.0% were females while 28.0% were males. Eighteen of them were removed from the study on account of incomplete data collection, making the actual number 375 subjects. Majority of the subjects were Igbos. The mean age of the subjects was 39 ± 11 years. For all the study subjects, the mean value of serum LDL was 2.05 ± 0.58mmol/l serum total cholesterol 4.26 ± 0.90mmol/l, serum triglyceride 1.23 ± 0.37mmol/l, and serum HDL 1.18 ± 0.39mmol/l. The mean values of other variables are shown in Table 1. The median value of the CD4 cells count was 371 cells/ml. Out of the 375 subjects, 323(82.2%) have desirable serum LDL (< 2.6mmol/l) whereas 69(17.6%) have borderline serum LDL (2.6-4.1mmol/l). None of the subjects has high serum LDL (> 4.1mmol/l). The number of subjects who have borderline serum total cholesterol (5.2-6.2mmol/l) were 39(9.9%) whereas 6(1.5%) have high serum total cholesterol (> 6.2mmol/l). Similarly, 30(7.6%) have borderline serum triglyceride (1.7-2.2mmol/l) whereas 9)2.3%) have high serum triglyceride (> 2.2mmol/l). Low serum HDL (< 1.0mmol/l) was observed in 135(34.4%) of the subjects whereas high or normal serum HDL was found in 358(65.5%). There was no significant association between BMI and dyslipidemia (p = 818) (Table 2). The association between dyslipidemia and CD4 cells count was significant (p = 0.027). Out of 323 subjects who have desirable serum LDL (2.6-4.1mmol/l), 35(10.8%) have CD 4 cells count < 200cells/ml, whereas CD4 > 200 cells/ml were observed in 15.8% of those who have borderline serum LDL. The prevalence of dyslipidemia increased as CD 4 cells count increased (Table 2 and Figure 1). No significant association was observed between dyslipidemia and ClCr (p = 0.579), 24HUP (p = 0.256) as well as 24HUOsm (p = 0.451) (Table 2). Conversely, a significant association was observed between dyslipidemia and anemia in these subjects (df = 3, p = 0.001) (Table 2). Out of 178 subjects who have Hb 10.0-12.0g/dl, 39(21.9%) have borderline serum LDL. Out of 6 subjects that have Hb < 7.0g/dl, 4(66.7%) have borderline serum LDL. This showed that the prevalence of dyslipidemia was high among the subjects who have severe anemia (Table 2 and Figure 2). There was no significant correlation between dyslipidemia and SCr, SUP, SUCr, SUOsm, ClCr, 24HUP, 24HUCr as well as 24HUOsm.

**Table 1:** Characteristics of variables in HIV subjects

Variables(mean±SD)	HIV Subjects
Body Mass Index (kg/m^2^)	26.2 ± 5.4
Hemoglobin (g/dl)	11.2 ± 1.8
SUOsm (mOsm/kgH_2_O)	464 ± 271
Spot Urine Protein(mg/dl)	11.89 ± 19.13
Spot Urine Creatinine (mg/dl)	137.21± 98.47
24-Hour Urine Protein (g)	0.187 ± 0.290
24-Hour Urine Creatinine (mg)	1507 ± 781
24HUOsmkgH_2_O	564 ± 501
Creatinine Clearance (mls/min)	91.42 ± 22.98

SD=standard deviation, SUOsm=spot urine osmolality, 24UOsm=24-hour urine osmolality,

**Table 2:** Distribution and characterization of variables with different levels of serum LDL (n=376)

VARIABLES	Desirable serum LDL (<2.6mmol/l) (n/%) N=110	Borderline serum LDL (2.6-4.1mmol/l) (n/%) N=20	ChiSquare	df	LHR	P value
BMI <18.5	21(87.5%)	3(12.5%)	0.932	3	0.811	0.818
18.5-24.9	110(82.1%)	24(17.4%)				
25.0-29.9	125(83.3%)	25(15.7%)				
≥30	67(79.8%)	17(20.2%)				
CD4 <200	35(10.8%)	14(20.8%)	4.874	1	0.045	0.027
>200	288(89.2%)	54(19.4%)				
ClCr mls/min?90	164(82.8%)	34(17.2%)	1.092	2	0.563	0.579
60-89	115(80.4%)	28(19.6%)				
30-59	29(87.4%)	4(12.1%)				
24HUP(mg)<300	208(81.2%)	48(18.8%)	4.047	3	0.277	0.256
300-3499	46(95.2%)	8(14.8%)				
**24HUOmsOm/kgH2O**						
<300	201(83.4%)	40(16.5%)	1.501	2	0.488	0.451
300-750	92(92.1%)	20(17.9%)				
>750	16(72.7%)	6)27.3%)				
Hb (g/dl)>12.0	110(87.3%)	16(12.7%)	15.978	3	0.004	0.001
10.0-12.0	139(78.1%)	31(29.1%)				
7.0-9.9	72(87.8%)	10(12.2%)				
<7.0	2(33.3%)	4(66.7%)				

LHR=Likelihood ratio, BMI=body mass index, ClCr=creatinine clearance, 24HUP=24-hour urine protein, 23HUOsm=24-hour urine osmolality, Hb=hemoglobin

## Discussion

The prevalence of elevated serum LDL (17.0%) found in this study is slightly higher than 11.2% reported by Manathu et al in treatment-naïve HIV subjects [22]. Their study also noted that the prevalence increased to 40.0% in cART-treated subjects [22]. However, another study showed a prevalence of 52.2% in a general clinic population who were HIV-seronegative [10]. Although dyslipidemia attributable to elevated serum LDL was comparatively high in both the general population [10] and HIV subjects on cARTs [22], treatment-naïve HIV subjects have clinically prevalent dyslipidemia, as shown in our study. Elevated serum triglyceride was prevalent in 9.9% of treatment-naïve HIV subjects in this study. Two studies, however, reported a higher prevalence of 22.5% and 28.0% [22, 23]. Both studies were conducted in east Africa, whereas ours was in Nigeria. Perhaps, there could be some racial, nutritional or other factors that might account for this difference. In contrast, a prevalence of 7.3% was reported in another study in the general clinic patients within the same domain as ours 10. This study observed, for low serum HDL, a prevalence of 34.0% which was much lower than 51.3% and 68.0% documented in two studies in treatment-naïve HIV subjects [22, 23]. This observed difference might be explained, in part, perhaps, by the difference in the time of presentation. Many of our study subjects responded to HIV awareness creation campaigns for early voluntary testing and counselling. However, a study in a general population reported a low serum HDL prevalence of 55.1%, a figure that is higher than what was observed in our study. The aggregate prevalence of dyslipidemia, when elevated serum LDL, elevated serum triglyceride and low serum HDL were put together, was high in this study, indicating the high degree of cardiovascular risks in treatment-naïve HIV subjects. In this study, significant association was observed between serum LDL and CD4 cells count. It was further observed that the prevalence of dyslipidemia increased as CD 4 cells count increased, similar to the observation in another study [24]. Reduced CD4 cells count is an index of suppressed immunity and, expectedly CD4 cells should increase with normal weight and obesity, but decline with underweight.

This study showed that the prevalence of dyslipidemia was high among the treatment-naïve HIV subjects who have severe anemia. One study found a direct positive correlation between hemoglobin and dyslipidemia [25]. The study, however, differed from our study: it assessed the impact of anemia on serum lipid in premenopausal women whereas our study subjects were treatment-naïve HIV subjects of both sexes and age range of 16-65 years. On the contrary, one study did not observe any significant correlation between serum LDL and anemia [25]. Iron stores at different levels found in premenopausal women and some agents that cause dyslipidemia, can influence different levels of hemoglobin [25]. Some causes of dyslipidemia can alter hemoglobin levels [25]. This study admittedly, did not evaluate the mechanism by which serum LDL is influenced in the setting of anemia. Serum creatinine is observed to rise as renal function declines. In addition, dyslipidemia has been documented in chronic kidney disease states [26]. There was a paucity of studies on the influence of dyslipidemia on SCr. This study, however, did not find any significant correlation between dyslipidemia and SCr. Both elevated SUP and abnormal 24HUP might be observed in proteinuric renal diseases irrespective of etiology [27]. Nephrotic proteinuria is associated with elevated LDL [28]. Both SUP and 24HUP did not show any significant correlation with dyslipidemia in this study, probably suggesting that our study subjects were not likely to have proteinuric renal disease. Excretion of creatinine in random urine samples is dependent on many factors including intake of protein, muscle mass, some medications and state of renal function, among others [18, 29]. However, this study did not find any significant correlation between dyslipidemia and SUCr as well as 24HUCr. Renal filtration function impairment, in the chronic phase, marked by declining ClCr might be associated with reduced serum LDL. This study also did not observe any significant correlation between dyslipidemia and ClCr. Some physiologic states, diseases and environment conditions have been reported to alter SUOsm and 24HUOsm in the general population [18]. In addition, some renal diseases especially with the involvement of the interstitial compartment might affect renal concentrating ability and subsequently alter SUOsm and 24HUOsm [30]. Our study did not observe any significant correlation between dyslipidemia and urine osmolality in treatment-naïve HIV subjects.

## Conclusion

The prevalence of dyslipidemia was high in the study subjects. Abnormal CD4 cells count and anemia were common in treatment-naïve HIV subjects who have dyslipidemia. There is a need for clinicians to routinely evaluate HIV subjects for dyslipidemia and further search for anemia and low CD4 cells count in those who have dyslipidemia in the early stage of the infection.

**Limitations**: The study population was small. A larger study size would have been more representative of the population.

### What is known about this topic

- HIV infection is prevalent in Nigeria;
- HIV might contribute to dyslipidemia with cART;
- Dyslipidemia in HIV has not been completely evaluated in Nigeria.

### What this study adds

- Dyslipidemia is prevalent in cART -naïve HIV subjects in Nigeria;
- Abnormal CD4 cells count and anemia were common in treatment-naïve HIV subjects who have dyslipidemia;
- There is a need for clinicians to routinely evaluate HIV subjects for dyslipidemia and further search for anemia and low CD4 cells count in those who have dyslipidemia in the early stage.

## Competing interests

The author declares no competing interests.
